# Ritonavir-boosted Nirmatrelvir and COVID-19 outcomes in the age of Omicron variant

**DOI:** 10.1097/MS9.0000000000000169

**Published:** 2023-02-17

**Authors:** Laiba Imran, Rooja Zubair, Sanila Mughal, Ramsha Shakeel

**Affiliations:** Department of Internal Medicine, Dow University of Health Sciences, Karachi, Pakistan

**Keywords:** coronavirus disease 2019, Nirmatrelvir, Omicron, Ritonavir

## Abstract

Nirmatrelvir boosted with Ritonavir is the recommended and preferred treatment for COVID-19. Because real-world evidence of Nirmatrelvir’s antiviral activity against the Omicron variation is minimal, our study focuses on recent papers suggesting the use of Ritonavir-boosted Nirmatrelvir in the real world against the most frequent SARS coronavirus variant circulating worldwide (Omicron). Despite sparse clinical evidence, we discovered that Ritonavir-boosted Nirmatrelvir reduced COVID-19-related hospitalization and mortality during the onset of the Omicron variant. Furthermore, this study discusses the main limitations and offers recommendations for administering this drug in non-hospitalized COVID-19 patients at high risk for severe infection.

## Introduction

Since the first diagnosed case of severe acute respiratory syndrome coronavirus (SARS-CoV-2) was reported on 17 November 2019^1^, the coronavirus disease 2019 (COVID-19) global outbreak has claimed an estimated 6.5 million lives, including 625 million confirmed cases worldwide^2^. The original SARS-CoV-2 virus had evolved, igniting numerous new variations. In an attempt to prioritize research and surveillance of several variants of the SARS-CoV-2 virus, WHO has categorized them into the following groups: variants under monitoring, variants of interest, and variants of concern (VOCs)^3^. Following the previous VOCs, including Alpha (B.1.1.7), Beta (B.1.351), Gamma (P.1), and Delta (B.1.617.2), on 26 November 2021, another variant named Omicron (B.1.1.529) was mentioned too as the new VOC, and it instantly raised worldwide concerns^3^,^4^. Since its designation as a VOC, the viruses constituting the Omicron complex have continued to evolve. As of October 2022, Omicron is currently the dominant circulating variant worldwide^3^.

Nonetheless, immunization remains one of the most significant advances against the COVID-19 pandemic^5^, individuals who still become infected with COVID-19 currently face limited options for treatment. To prevent their progression to hospitalization or death, several antiviral drugs have been investigated or approved, namely remdesivir, molnupiravir, and Nirmatrelvir/Ritonavir^6^,^7^. Remdesivir, an adenosine analog, is the only treatment for COVID-19 permitted by the Food and Drug Administration (FDA). In nonhospitalized COVID-19 patients considered to have a significant risk for the progression of disease severity, remdesivir is advised to be injected for three days^7^,^8^. While remdesivir requires intravenous administration, the FDA promulgated an Emergency Use Authorization for an oral inhibitor of RNA-dependent RNA polymerase, molnupiravir, on 23 December 2021^9^. However, this ribonucleoside antiviral agent is associated with the alarming possibility of inducing mutations in human DNA^10^. Among these drugs, Ritonavir-boosted Nirmatrelvir remains the recommended and preferred treatment for COVID-19^7^.

Nirmatrelvir is a SARS-CoV-2 protease (nsp5 protease/M) inhibitor that can be taken orally. Inhibition of this enzyme renders the virus incapable of processing polyprotein precursors into functional units, preventing viral replication^11^,^12^. In order to bring Nirmatrelvir serum concentrations up to the desired therapeutic range and further extend the half-life of this antiviral drug, it is necessary to combine it with Ritonavir, a potent cytochrome P450 3A4 inhibitor. Their combination is referred to as Paxlovid^13^. The following are guidelines for using Nirmatrelvir and Ritonavir together^14^: patients having mild to moderate infection of COVID-19 within the initial 5 days of the appearance of symptoms, and having a significantly higher risk of progression into severe form^15^. Paxlovid has been shown in a number of studies to decrease hospitalization and mortality in COVID-19 patients with mild to moderate infections.

The EPIC-HR, a randomized, double-blind, placebo-controlled trial, served as the foundation for the Emergency Use Authorization’s approval of Ritonavir-boosted Nirmatrelvir^16^. This study included 2246 adult nonhospitalized individuals with a confirmed COVID-19 infection who had not been immunized against SARS-CoV-2. All of them were predisposed to a single risk factor associated with developing a severe COVID-19 infection. For 5 days, Nirmatrelvir and Ritonavir (300 and 100 mg, respectively) were administered twice daily. On day 28, it showed an 88% decrement in COVID-19-related hospitalizations with no deaths being reported. In contrast to the Nirmatrelvir/Ritonavir group, the placebo group did report 12 deaths, with Delta being the predominant SARS-CoV-2 variant in both groups. However, the real-world evidence of an antiviral effect of Nirmatrelvir against the Omicron variant is limited. An observational, retrospective cohort study^17^ recruited 109 254 adult patients, aged 40 years or older, with SARS-CoV-2 infection. It was determined that they had a significant risk of developing a severe disease. Among these 109 254 individuals, 3902 were treated with Nirmatrelvir. In this study, patients over the age of 65 who received Nirmatrelvir treatment had significantly lower rates of hospitalization and death. It is to be noted that this study was conducted during the peak era of the Omicron variant. Additionally, an Italian study^18^ recruited 257 adults who tested positive for the SARS-CoV-2 virus and had at least one condition or comorbidity associated with an increased risk for severe infection. The results demonstrated that in the Omicron period, particularly in vaccinated patients, Nirmatrelvir/Ritonavir therapy remains successful in decreasing hospitalization rates and mortality related to COVID-19 infection. A Hong Kong study^19^ conducted during the Omicron wave also reported that early initiation of Nirmatrelvir/Ritonavir in nonhospitalized COVID-19 patients was associated with a low risk of mortality, hospitalization, and in-hospital disease progression.

As a result, these studies demonstrated that Paxlovid is expected to be effective against various strains of the SARS-CoV-2 virus, including the Omicron variant. However, before prescribing this Ritonavir/Nirmatrelvir regimen, physicians should thoroughly evaluate the concomitant medications, such as recreational drugs or herbal supplements, since clinically significant drug-drug interactions might be caused by Ritonavir-boosted Nirmatrelvir^20^. In addition, the drug’s long-term safety profile and efficacy remain unknown, drawing attention to potential future studies and trials. Moreover, there is little or no data on using Nirmatrelvir in conjunction with anti-SARS-CoV-2 monoclonal antibodies or various antiviral therapies for the treatment of COVID-19 patients. Further clinical trials are required to determine whether Nirmatrelvir-associated combination therapy can be used to treat or prevent severe infections. In addition, pregnant or breastfeeding women were excluded from different clinical trials, such as the EPIC-HR. Additionally, there is a dearth of information regarding the effectiveness of longer courses or a second course of this drug regimen and the treatment duration in severely immunocompromised patients. Despite limited clinical evidence, all available data suggest the real-world effectiveness of Nirmatrelvir/Ritonavir in preventing hospitalization and death related to COVID-19 during the emergence of the Omicron variant. We urge the authorities to conduct the necessary research to ensure our expectations are realized. In conclusion, we are hopeful that using Ritonavir-boosted Nirmatrelvir or Paxlovid for COVID-19 infection, together with behavioral modifications to prevent exposure to SARS-CoV-2 and vaccine programs, may change the course of the COVID-19 pandemic in the age of the Omicron variant.

## Ethical approval

This article did not involve patients; therefore, no ethical approval was required.

## Consent

This study was not done on patients or volunteers; therefore, no written consent was required.

## Sources of funding

There was no source of funding for this article.

## Author contribution

L.I.: conception of the study, drafting of the work, final approval, and agreeing to the accuracy of the work. R.Z.: conception of the study, drafting of the work, final approval, and agreeing to the accuracy of the work. S.M.: conception of the study, drafting of the work, final approval, and agreeing to the accuracy of the work. R.S.: critical revision of the manuscript, edit, final approval, and agreeing to the accuracy of the work.

## Conflict of interest disclosure

There was no conflict of interest.

## Research registration unique identifying number (UIN)

1. Name of the registry: not applicable

2. Unique Identifying number or registration ID: not applicable

3.Hyperlink to your specific registration (must be publicly accessible and will be checked): not applicable.

## Guarantor

Laiba Imran, Ramsha Shakeel.

## Provenance and peer review

Not commissioned, externally peer-reviewed.
